# The Effect of Different Types of Adhesive Systems on Marginal Integrity of Class II Resin Composite Restoration

**DOI:** 10.3290/j.jad.c_2789

**Published:** 2026-08-21

**Authors:** Phattarasinee Phuntusuntorn, Kulapatch Engkatanachai, Weerachai Singhatanadgit, Yanee Tantilertanant

**Affiliations:** a Phattarasinee Phuntusuntorn Residency training program in Restorative and Operative Dental Care, Department of Operative Dentistry, Faculty of Dentistry, Chulalongkorn University, Henri-Dunant Road, Pathumwan, Bangkok 10330, Thailand. Investigation, writing (original draft), read and approved the final version of the manuscript.; b Kulapatch Engkatanachai Researcher, Department of Operative Dentistry, Faculty of Dentistry, Chulalongkorn University, Henri-Dunant Road, Pathumwan, Bangkok 10330, Thailand. Writing (review and editing), validation, read and approved the final version of the manuscript; c Weerachai Singhatanachai Associate Professor, Faculty of Dentistry and Research Unit in Mineralized Tissue Reconstruction, Thammasat University (Rangsit Campus), Pathum Thani 12121, Thailand. Writing (review and editing), validation, read and approved the final version of the manuscript.; d Yanee Tantilertanant Assistant Professor, Department of Operative Dentistry, Faculty of Dentistry, Chulalongkorn University, Henri-Dunant Road, Pathumwan, Bangkok 10330, Thailand. Supervision, writing (review and editing), validation, read and approved the final version of the manuscript.

**Keywords:** cervical margin relocation, deep margin elevation, microleakage, universal adhesive.

## Abstract

**Purpose:**

To evaluate the marginal integrity of cervical margin relocation (CMR) or deep margin elevation (DME) restorations using a universal adhesive in etch-and-rinse and self-etch modes, compared to gold-standard systems after 6-month water aging.

**Methods and Materials:**

Standardized mesial and distal Class II cavities were prepared in 32 human maxillary premolars. Specimens were assigned to four groups according to adhesive systems and modes: OFL (OptiBond FL), CSE (Clearfil SE Bond), UER (Single Bond Universal; etch-and-rinse), and USE (Single Bond Universal; self-etch) (n = 16 per group). After DME/CMR and final restoration, specimens were stored in 37°C distilled water for 6 months. Marginal integrity was assessed qualitatively via micro-CT by two calibrated observers and quantitatively via 0.5% methylene blue dye. Data were analyzed using weighted kappa coefficient, Wilcoxon signed-rank test, two-way repeated measures analysis of variance (ANOVA), and one-way ANOVA with Games-Howell post-hoc tests (α = 0.05).

**Results:**

Micro-CT showed high inter-examiner reliability (κ = 0.84) and stable interfacial adaptation across groups post-aging. However, dye penetration revealed that adhesive mode significantly affected microleakage (*p* < 0.05), while proximal box concavity geometry had no significant effect (*p* > 0.05). The USE group exhibited the lowest mean microleakage (0.94 ± 0.62 mm), showing no significant differences from OFL and CSE. The UER group showed the highest mean microleakage (1.48 ± 0.09 mm), which was significantly higher than the USE group (*p* < 0.05).

**Conclusion:**

Within the limitations of this study, the self-etch application of universal adhesives provided superior durability of marginal seals for DME compared to the etch-and-rinse mode. Optimizing matrix application also minimized the influence of concavity geometry.

The minimally invasive approach is fundamental to contemporary restorative dentistry,^25^ prioritizing the preservation of tooth structure to maintain oral function and enhance the patient’s quality of life.^36^ While this conservative concept is ideal, its clinical application becomes increasingly challenging in cases of severe tooth loss. A major complication arises when pathological destruction causes cervical margins to extend deep into the subgingival area.^12^ Traditionally, managing these deep defects has required invasive clinical procedures, such as surgical crown lengthening, surgical extrusion, or orthodontic forced eruption, to preserve the healthy biological condition and provide adequate accessibility for the effective restorative procedure.^30^ However, these interventions are often limited, particularly in elderly patients with anatomical constraints or compromised physiological health.^21^ To overcome these limitations, deep margin elevation (DME) or cervical margin relocation (CMR) has been introduced as a more conservative alternative.^40^


Despite the clinical advantages of DME, the long-term success is critically dependent on the integrity of the dentin–adhesive interface at the cervical margin. This procedure inherently involves bonding to cervical or root dentin, which is considered among the most challenging substrates for adhesive procedures.^38^ The cervical dentin region often presents with sclerotic dentin, a highly mineralized and acid-resistant substrate that can hinder effective resin monomer infiltration.^34^ Furthermore, the intrinsic quality of root dentin, characterized by a different tubule orientation and lower mineral content compared to coronal dentin, combined with the constant proximity to gingival crevicular fluid, creates a highly sensitive environment for adhesion. The anatomical variations of root concavity in posterior teeth also impede a hermetic cervical seal, hampering the optimal bond quality. These factors prone to compromise long-term bond durability and the integrity of the adhesive interface.^38^ Therefore, it is highly worthwhile to investigate bonding protocols that can ensure a durable and reliable seal in this demanding area.

In response to these substrate challenges, the commercial availability of dental adhesives has evolved significantly, with continuous improvements focusing on simplifying clinical procedures while giving high versatility.^39^ Despite these advancements, addressing a consensus strategy for bonding to the deep cervical area encountered in DME remains a critical clinical objective. Gold-standard multi-step adhesives OptiBond FL (Kerr; Brea, CA, USA) and Clearfil SE Bond (Kuraray Noritake Dental; Tokyo, Japan) are recognized for their ability to provide predictable and durable adhesive interfaces, demonstrating consistent performance especially on complicated root dentin.^10,26
^ However, the technical sensitivity of multi-step protocols in areas with limited accessibility has led to the increasing use of universal adhesives. By accommodating various bonding protocols according to operator preference, universal adhesives exhibited satisfactory efficacy across diverse applications.^13^ Although universal adhesives provide clinical convenience, their ability to maintain a long-term marginal seal in the specific context of DME remains a point of significant divergence in the literature. Evidence suggested that the chemical simplification of these systems increased the hydrophilicity of the adhesive interface, rendering them more susceptible to hydrolytic degradation over time compared to gold-standard systems.^6,8
^ Conversely, recent clinical data indicated that universal adhesives, when utilized in self-etch mode, demonstrated marginal staining at the gingival interface comparable to that of gold-standard self-etch protocols.^9^ Consequently, a definitive consensus on the optimal strategy for bonding to DME cavities remains unclear.

Taken together, these factors warrant a comprehensive investigation of adhesive strategies to identify the most effective approach for maintaining a marginal seal at the cervical interface. Therefore, the purpose of this study was to evaluate the marginal integrity of DME restorations using a universal adhesive in both etch-and-rinse and self-etch modes, compared with traditional gold-standard systems, aging with 6-month water storage. The hypotheses tested were that: (1) the type of adhesive systems would affect the marginal integrity at the gingival margin of Class II resin composite restorations; and (2) the proximal box concavity geometry would have a significant effect on the marginal integrity at the gingival margin in these restorations.

## Methods and Materials

### Specimen Preparation and Cavity Design

Following institutional ethical approval and patient informed consent, 32 intact permanent human maxillary premolars, extracted for orthodontic reasons, were selected, cleaned, and examined under a stereomicroscope at 20× magnification (SZ 61, Olympus, Japan) to exclude teeth with caries, cracks, previous restorations, or enamel defects. The specimens were stored in 1% chloramine-T solution at 4°C for 1 week and immersed in distilled water until use.

The flowchart of experimental design and procedures is depicted in Figure 1. To standardize the preparations following a modified protocol,^31^ the occlusal cusps and proximal contours were flattened using a dental model trimmer (Dentalfarm, Italy) to achieve a total anatomical crown height of 5 mm, measured from 1 mm apical to the proximal cementoenamel junction (CEJ). Standardized Class II proximal cavities (OM and OD) were prepared with dimensions of 3 mm buccolingual width, 1.5 mm mesiodistal depth, and 5 mm occluso-gingival height) using 1.0 mm medium-grit cylindrical diamond burs (No. 841 010, Meisinger, Germany), which were replaced every five preparations. Each specimen was then mounted in a polyvinyl chloride (PVC) mold and stabilized with silicone putty and polyvinyl siloxane material (Betasil, Müller-Omicron, Germany) to simulate the gingival sulcus, which was positioned approximately 1 mm apical to the CEJ. All samples were prepared by one operator (PP).

**Fig 1 Fig1:**
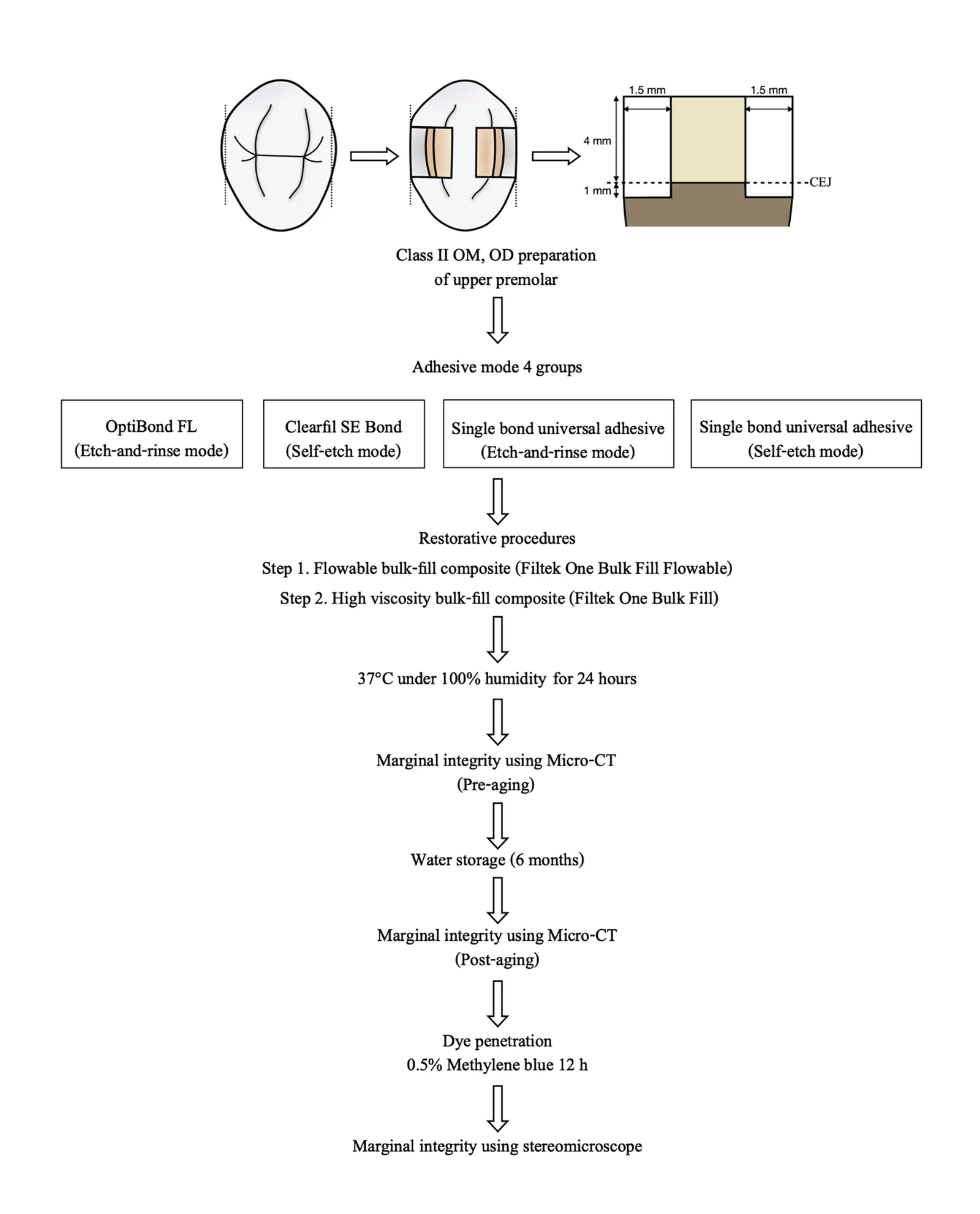
A flow chart of the experimental procedures.

### Grouping and Restorative Procedures

All prepared teeth were randomly allocated into four experimental groups (n = 8 teeth per group, providing 16 proximal cavities per group) based on the adhesive system and application mode: OFL (OptiBond FL; etch-and-rinse), CSE (Clearfil SE Bond; self-etch), UER (Single Bond Universal; etch-and-rinse), and USE (Single Bond Universal; self-etch). The chemical composition of the materials used in this study is shown in Table 1.

**Table 1 table1:** Chemical composition of materials used in this study

Material	Composition	pH	Manufacturer	Batch no.
OptiBond FL	Primer: HEMA, GPDM, PAMM, ethanol, water, photoinitiator Adhesive: TEGDMA, UDMA, GPDM, HEMA, bis-GMA, filler, photoinitiator	1.8	Kerr; Brea, CA, USA	Lot 9651407 REF 26684
Clearfil SE Bond	Primer: 10-MDP, HEMA, Hydrophilic dimethacrylate, camphorquinone, water Adhesive: 10-MDP, bis-GMA, HEMA, hydrophobic dimethacrylates, camphorquinone, colloidal silica	1.9	Kuraray Noritake Dental; Tokyo, Japan	Lot 000094 REF #1970-TH
Single Bond Universal Adhesive	Adhesive: 10-MDP, Vitrebond copolymer, HEMA, dimethacrylate resins, filler, silane, initiator, ethanol, water	2.7	Solventum; St. Paul, MN, USA	Lot 10510365 REF 41282
Gel Etchant	37.5% phosphoric acid, silica thickener		Kerr; Brea, CA, USA	Lot 9651407 REF 26684
Filtek One Bulk Fill (A2)	Monomer matrix: AUDMA, AFM, 1,12-dodecane-DMA, and UDMAFillers: Combination of zirconia/silica, ytterbium trifluoride, Filler loading is about 76.5% by weight (58.4% by volume)		Solventum; St. Paul, MN, USA	Lot 9533868 REF 4869A2
Filtek One Bulk Fill Flowable (A2)	Monomer matrix: Bis-GMA, UDMA, BisEMA, and Procrylate resins Fillers: Combination of zirconia/silica, ytterbium trifluoride, Filler loading is about 64.5% by weight (42.5% by volume)		Solventum; St. Paul, MN, USA	Lot 9533442 REF 4862A2
Bis-GMA, bisphenol A diglycidyl ether dimethacrylate; HEMA, 2-hydroxyethyl methacrylate; TEGDMA, triethylene glycol dimethacrylate; 10- MDP, 10-methacryoloyloxydecyl dihydrogen phosphate; UDMA, urethane dimethacrylate; GPDM, glycerol phosphate dimethacrylate; PAMM, phthalic acid monoethyl methacrylate; Bis-EMA, ethoxylated bisphenol A glycol dimethacrylate.				

All bonding procedures followed the manufacturers’ instructions, as detailed in Table 2. The proximal cavities were restored sequentially; while one cavity was undergoing the restorative procedure, the contralateral side was protected with thin aluminum foil to prevent accidental light exposure. All materials were light-polymerized using a light-emitting diode (LED) curing unit (Bluephase; Ivoclar Vivadent, Schaan, Liechtenstein) with an irradiance of 1,200 mW/cm^2^.

**Table 2 Table2:** Adhesive systems, resin composites, and their application protocols

Materials	Bonding steps recommended by manufacturer	Restorative techniques of resin composite	Group code
OptiBond FL	Etch: Apply etchant 15 s, rinse with water 15 s, gently air dry 3 s Prime: Apply primer with light scrubbing motion for 15 s, gently air dry 5 s Bond: Apply a thin coat of bonding agent and light cure for 20 s	Filtek One Bulk Fill Flowable 1-mm on gingival floor, light cure for 20 s and covered by 4-mm increments of Filtek One Bulk Fill and light cure for 40 s of each layer	OFL
Clearfil SE Bond	Prime: Apply a layer of primer, wait 20 s, gently air dry Bond: Apply bonding agent, remove excess with a light jet of air and light cure for 10 s	Filtek One Bulk Fill Flowable 1-mm on gingival floor, light cure for 20 s and covered by 4-mm increments of Filtek One Bulk Fill and light cure for 40 s of each layer	CSE
Single Bond Universal Adhesive	*Etch-and-rinse mode* Etch: Apply etchant 15 s, rinse with water 15 s, gently air dry 3 s Bond: Apply adhesive and rub for 20 s, dry gently for about 5 s, light cure for 10 s *Self-etch mode* Bond: Apply adhesive and rub for 20 s, dry gently for about 5 s, light cure for 10 s	Filtek One Bulk Fill Flowable 1-mm on gingival floor, light cure for 20 s and covered by 4-mm increments of Filtek One Bulk Fill and light cure for 40 s of each layer Filtek One Bulk Fill Flowable 1-mm on gingival floor, light cure for 20 s and covered by 4-mm increments of Filtek One Bulk Fill Flowable and light cure for 40 s of each layer	UER USE


#### First stage: DME

In the first stage, the deep proximal margins were elevated using a circumferential matrix system (Margin Elevation Band; Garrison Dental Solutions, Spring Lake, MI, USA) stabilized with a wooden wedge and polytetrafluoroethylene (PTFE) tape to ensure a tight cervical seal. Following the assigned adhesive protocol, a 1-mm layer of flowable bulk-fill composite (Filtek One Bulk Fill Flowable; Solventum; St. Paul, MN, USA shade A2) was placed on the gingival floor and light-cured for 20 s. After matrix removal, any excess material was refined with a no. 12 scalpel blade. The 1-mm elevation height was verified using a periodontal probe; any discrepancy was adjusted with a 1.0 mm medium-grit cylindrical diamond bur to ensure a standardized height.

#### Second stage: final restoration

In the second stage, a Tofflemire matrix system (Kerr, Brea, CA, USA) was positioned and stabilized with a wooden wedge. The adhesive procedure was repeated according to each group’s protocol. The remaining cavity was filled with a single 4-mm increment of high-viscosity bulk-fill composite (Filtek One Bulk Fill; Solventum; St. Paul, MN, USA, shade A2) and light-polymerized for 40 s. To ensure procedural consistency, all matrix bands were single-use.

Following matrix removal, the occlusal surfaces were finished and polished with aluminum oxide discs (Sof-Lex; Solventum; St. Paul, MN, USA). The restored teeth were then stored in an incubator at 37°C and 100% relative humidity for 24 h to simulate oral conditions before the following investigation was performed.

### Artificial Aging Process

To simulate the intraoral environment, the specimens were immersed in distilled water at 37°C for 6 months and kept in an incubator (Contherm 160M; Contherm Scientific, Lower Hutt, New Zealand). In accordance with ISO/TS 11405, the water was replaced every 7 days to maintain stable storage conditions.

### Evaluation of Marginal Integrity

#### Microcomputed tomographic (micro-CT) analysis

All restored teeth were subjected to micro-CT analysis to evaluate the restoration–tooth interface at two time points: pre-aging (baseline) and post-aging (after 6 months). Each specimen was fixed in a custom 10-mm diameter holder and scanned individually using a micro-CT scanner (Model 1273; SkyScan, Bruker, Kontich, Belgium). The X-ray source was operated at a voltage of 115 kVp and a current of 90 μA with a 0.3 mm copper filter, a resolution of 972 × 1536 pixels, and a voxel size of 10 µm. The scanning parameters were set as follows: a 360° rotation with a 0.4° rotation step and an exposure time of 1134 ms. The acquired two-dimensional projection images were subsequently converted into three-dimensional (3D) image stacks using NRecon reconstruction software (version 2.2.0.6; Bruker, Kontich, Belgium). To optimize image quality and minimize scanning artifacts, the reconstruction parameters were precisely adjusted, utilizing a beam-hardening correction of 20% and a ring artifact reduction factor of 8 to ensure clear visualization of the interfaces.

To evaluate interfacial adaptation before and after aging, the reconstructed micro-CT datasets from pre-aging and post-aging were co-registered, aligned, and digitally merged using the 3D image registration protocol within DataViewer software version 1.7.0.1 (Bruker, Kontich, Belgium) to ensure that identical cross-sectional planes were compared between timepoints. Multi-dimensional evaluations were performed across three distinct planes (coronal, sagittal, and transaxial) to compare point-to-point structural changes at the exact same tooth–restoration interfaces before and after the 6-month aging challenge. The qualitative assessment of structural integrity and the presence of interfacial gaps or micro-voids at the adhesive layer of the gingival margin was performed on the middle slice of each specimen. Evaluation was performed by two examiners who were independently calibrated prior to the study using a set of reference micro-CT images representing each score category. Both examiners were blinded to the group allocation (restorative material/technique) and timepoint identity of each specimen throughout the evaluation.^14,15,32
^ Datasets were presented in randomized order. Each examiner independently assessed the resin–dentin interface at each timepoint and assigned a score following a binary scoring system (0 = no change; 1 = detectable interfacial change after aging). Following independent scoring, inter-examiner agreement was quantified using the weighted kappa coefficient (κ). Discrepancies between examiners were resolved by consensus review under standardized monitor brightness and contrast settings.

### Dye Penetration Assessment

After all non-destructive investigations were performed, the aged specimens were prepared for dye penetration testing to evaluate the marginal integrity of the adhesive layer at the tooth–restoration interface. The root apices were sealed with sticky wax, and the entire tooth surface was coated with two layers of water-resistant nail varnish (Revlon; New York, NY, USA), leaving a window of 3 × 3 mm^2^ exposed at the gingival margin of the restoration. After the nail varnish had dried, the specimens were immersed in a 0.5% methylene blue solution for 12 h,^18^ then rinsed thoroughly under running water for 5 min. The teeth were embedded in acrylic resin. Using a low-speed diamond saw (Isomet 1000; Buehler, NY, USA) under water cooling, each tooth was sectioned buccolingually to separate the mesial and distal restorations, and then sectioned mesiodistally into three equal segments through the longitudinal axis.

All sections were examined under a stereomicroscope at 20× magnification (ML 9300; Meiji Techno, Saitama, Japan). Dye penetration was measured linearly from the outermost gingival margin to the innermost point of infiltration, with values ranging from 0 to 1.5 mm. Standardized digital images were captured, and the analysis was performed using OLYMPUS cellSens Standard imaging software (Olympus, Tokyo, Japan). For each restoration, the dye penetration values from the three segments were averaged to determine the mean microleakage score.

### Statistical Analysis

All data were analyzed with statistical software (SPSS version 30.0; IBM, Armonk, NY, USA), divided into two parts, including micro-CT data and data from dye penetration measurement.

Micro-CT analysis was used for qualitative evaluation of the restoration–tooth interface. Data were assessed for normality using the Shapiro–Wilk test. Marginal integrity scores, representing binary paired data from the same specimens at pre-aging and post-aging timepoints, were analyzed using the Wilcoxon signed-rank test for paired comparisons. Inter-examiner reliability was evaluated using the weighted kappa coefficient (κ), with values interpreted as follows: κ < 0.20 = slight agreement; 0.21–0.40 = fair; 0.41–0.60 = moderate; 0.61–0.80 = substantial; 0.81–1.00 = almost perfect agreement.^19^ The distribution of scores at pre-aging and post-aging timepoints was compared and reported descriptively with inferential statistics.

The mean dye penetration distance (0–1.5 mm) served as the principal measure of microleakage. To evaluate the effects of both adhesive mode and proximal box concavity geometry (mesial versus distal), a two-way repeated measures analysis of variance (ANOVA) followed by post-hoc analysis was performed. Additionally, differences among the four experimental groups were analyzed using one-way ANOVA followed by the Games–Howell post-hoc test for multiple pairwise comparisons. The level of significance was set at α = 0.05 for all analyses.

## Results

### Micro-CT Analysis

According to rigid 3D co-registration and superimposition of the pre-aging and post-aging micro-CT datasets, no macroscopically detectable volumetric differences were observed at the resin–dentin interface between timepoints across any experimental group. Meanwhile, dual-observer qualitative assessment was conducted by first validating the inter-examiner reliability. Inter-examiner reliability for qualitative interfacial integrity scoring was excellent, with a weighted Cohen’s kappa coefficient of κ = 0.84 (*p* < 0.001). Representative micro-CT images, as shown in Figure 2, demonstrated continuous interfacial adaptation at the dentin–restorative interface across all experimental groups at pre-aging and post-aging timepoints. No gross debonding, bulk voids, or catastrophic interfacial failure was detected in any specimen. Regarding the adhesive interface appearance, the OFL group exhibited the most perceptible radiolucent line at the resin–dentin interface among all groups evaluated, consistent with a thicker adhesive layer as anticipated given the material’s application characteristics. The USE and UER groups each presented a thin but consistently discernible radiolucent layer along the adhesive interface, reflecting a measurable though clinically contained interfacial zone. In contrast, the CSE group demonstrated a virtually imperceptible interface on micro-CT evaluation. Longitudinal comparisons between pre-aging and post-aging observations, performed on co-registered middle cross-sectional slices across all three orthogonal planes, revealed no statistically significant change in qualitative interfacial integrity scores in any experimental group following the 6-month water aging period (*p* > 0.05 for all groups). Figures 2a and 2b depict the representative micro-CT images of each group at pre-aging and post-aging timepoints, respectively.

**Fig 2a and b Fig2aandb:**
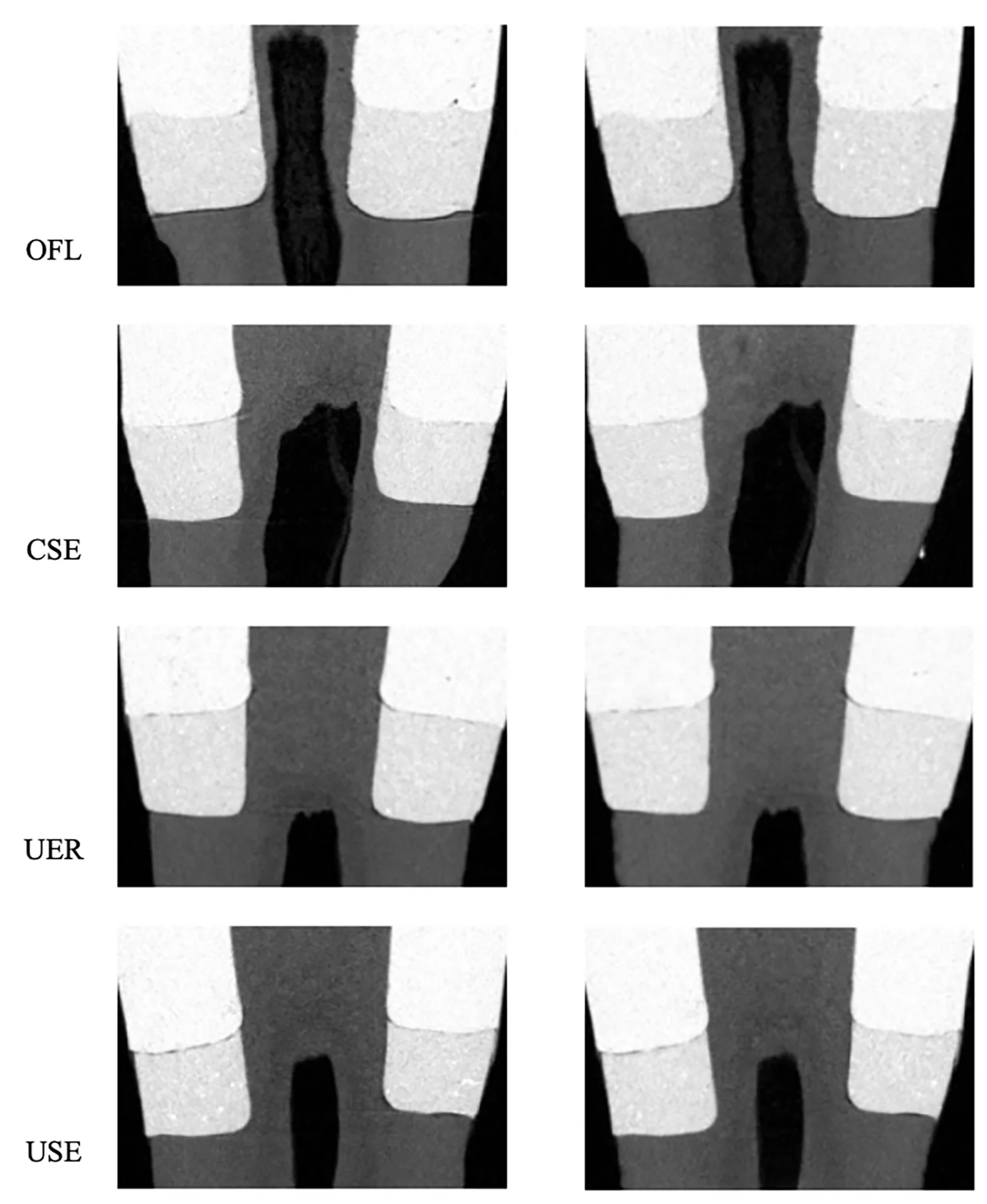
Representative micro-CT images of the tooth–restoration interface for all experimental groups: OFL, CSE, UER, and USE. Images are shown at pre-aging (a) and post-aging: 6-month water storage (b).

### Microleakage Evaluation (Dye Penetration)

The means and standard deviations of microleakage values across all experimental groups are presented in Table 3 and Figure 3. Two-way repeated measures ANOVA revealed that the adhesive application mode exerted a statistically significant effect on microleakage scores (*p* < 0.05). In contrast, neither the proximal box positions (mesial versus distal) nor the interaction term between adhesive mode and proximal box concavity geometry reached statistical significance (*p* > 0.05).

**Table 3 Table3:** Mean and standard deviation (SD) of microleakage values (mm) for the different adhesive modes and proximal positions

Adhesive mode	Group	Position		Total* (n = 64)
		Mesial (n = 32)	Distal (n = 32)	
OptiBond FL (Etch-and-rinse mode)	OFL	1.34 ± 0.44 ^ab,A^	1.42 ± 0.21 ^ab,A^	1.38 ± 0.34 ^ab^
Clearfil SE Bond (Self-etch mode)	CSE	1.11 ± 0.61 ^ab,A^	1.03 ± 0.65 ^ab,A^	1.07 ± 0.61 ^ab^
Single Bond Universal Adhesive (Etch-and-rinse mode)	UER	1.50 ± 0.00 ^a,A^	1.46 ± 0.13 ^a,A^	1.48 ± 0.09 ^a^
Single Bond Universal Adhesive (Self-etch mode)	USE	0.94 ± 0.64 ^b,A^	0.94 ± 0.65 ^b,A^	0.94 ± 0.62 ^b^
Different superscript small letters indicate statistically significant differencse among adhesive modes in the same position, analyzed by two-way repeated ANOVA followed by post-hoc analysis.Different superscript capital letters indicate statistically significant differences between positions in the same adhesive mode, analyzed by two-way repeated ANOVA followed by post-hoc analysis.*Different superscript small letters indicate statistically significant differences among adhesive modes, analyzed by one-way ANOVA followed by Games-Howell post-hoc test. *p* value < 0.05				

**Fig 3a and b Fig3aandb:**
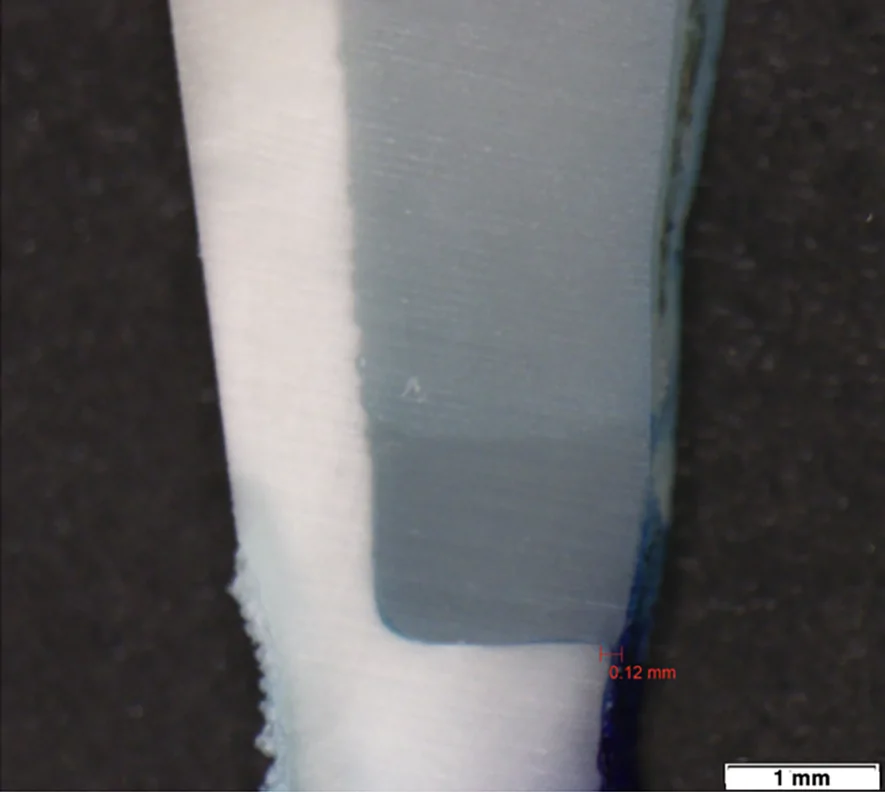

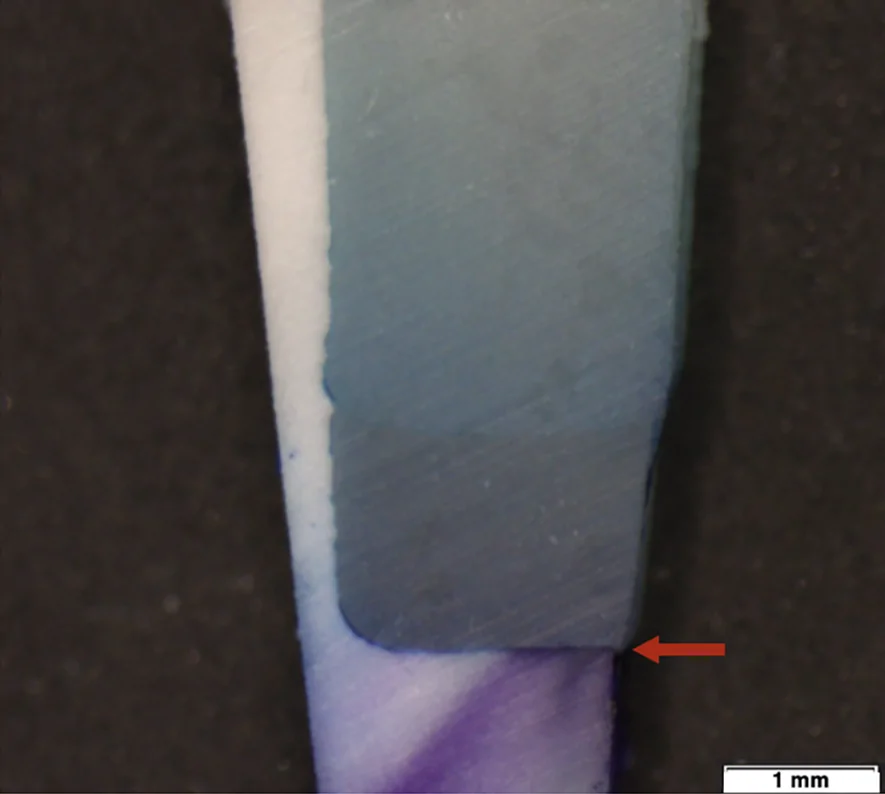
Representative stereomicroscope images of dye penetration. (a) Specimen showing no dye penetration at the interface. (b) Specimen showing microleakage (1.5 mm), with red arrows indicating dye penetration throughout the interfacial space between the tooth and restoration.

Regarding the adhesive mode and technique, one-way ANOVA followed by the Games–Howell post-hoc test revealed that the USE group exhibited the lowest mean values of microleakage (0.94 ± 0.62 mm). This value showed no statistically significant differences from the mean values of the OFL and CSE groups. However, the UER group showed the highest mean microleakage (1.48 ± 0.09 mm), which was significantly higher than that of the USE group (*p* < 0.05) (Table 3).

## Discussion

The present study investigated the effects of different types of adhesive systems and proximal box concavity geometry on the marginal integrity of Class II DME resin composite restorations following hydrolytic aging via water storage. The principal finding demonstrated that a universal adhesive applied in self-etch mode conferred superior marginal sealing quality to the prepared cavity wall compared with the etch-and-rinse application mode. Furthermore, variation in proximal box concavity geometry did not compromise marginal integrity when appropriate matrix system application was employed. Therefore, the hypotheses were partially rejected.

The difficulty of achieving a durable bond to the cervical margin, particularly in cases involving DME, has remained a primary concern in restorative dentistry. The findings indicated that the self-etch approach with either multi-step or simplified universal adhesives maintained acceptable marginal seals even after exposure to hydrolytic aging. This performance is likely attributable to the mild etching aggressiveness, which prevents total demineralization and limits the exposure of the collagen matrix.^3^ By retaining a layer of hydroxyapatite on the collagen surface, the adhesive system provides a protective sheath that mitigates the risk of hydrolytic degradation and subsequent bond failure.^28,44
^


Furthermore, the mineral left behind in the complex dentinal structure provided the necessary calcium ion source for interaction with functional monomers. Specifically, the 10-methacryloyloxydecyl dihydrogen phosphate (10-MDP) monomer reacts with the residual hydroxyapatite to form stable, insoluble MDP-Ca salts. This chemical interaction, in conjunction with micromechanical interlocking, substantially increases the hybrid layer’s resilience against environmental and hydrolytic degradation,^43,44
^ and occurs during long-term water storage. The structural integrity of the hybrid layer is further reinforced by the inclusion of a polyalkenoic acid copolymer (Vitremer copolymer). This component provides additional carboxyl groups for ionic bonding with calcium.^20^ The presence of this specific copolymer has been shown to enhance moisture tolerance and may ensure a stable chemical bond to the tooth substrate even under suboptimal conditions.^22^


In contrast, the highest microleakage values observed in the UER group were likely attributable to the aggressive demineralization of the dentin substrate by phosphoric acid. A previous study indicated that both deep coronal dentin and radicular dentin exhibit heightened susceptibility to phosphoric acid demineralization relative to milder self-etch primers.^4^ Specifically, phosphoric acid treatment resulted in significantly wider dentinal tubule openings,^4^ presenting a morphological impediment to complete resin infiltration. This discrepancy left unprotected collagen fibers at the bottom of the hybrid layer, which are highly susceptible to hydrolytic and enzymatic degradation.^6,38
^


Moreover, the characteristically thin adhesive interface produced by universal adhesives possesses a limited capacity for interfacial stress dissipation. This nature is particularly critical within the high C-factor of a proximal box, where shrinkage stress is maximized.^17,37
^ The presence of residual hydrophilic components within such a thin layer may further inhibit the degree of conversion, especially when applied to the deeply demineralized and inherently moist radicular dentin substrate.^16,33
^ Collectively, these morphological and chemical deficiencies contribute to the inferior marginal integrity observed when universal adhesives were utilized in etch-and-rinse mode.

The three-step etch-and-rinse gold standard (OFL) provides a substantial film thickness, probably seen as a radiolucent line in micro-CT (Fig 2), and enhanced stress-buffering capabilities, attributed to its separated highly filled hydrophobic bonding, acting as an elastic buffer zone capable of absorbing the polymerization shrinkage stress generated during composite restoration.^7,17,37
^ However, the microleakage length, which was greater than that of the self-etch approach, yet not significant, suggested the high degree of technique sensitivity inherent in deep proximal box configurations remains a decisive clinical variable. The utilization of an etch-and-rinse (ER) protocol in the humid environment of a deep proximal cervical or radicular dentin is characterized by a narrow therapeutic window. It is notably less clinically forgiving than self-etching alternatives. Achieving an optimal hybrid layer in these regions requires meticulous moisture control and rigorous application protocols to prevent collagen collapse. Consequently, the complexity of the ER approach in challenging anatomical sites, such as the deep proximal box, underscores the necessity for simplified yet resilient adhesive strategies that reduce the risk of operator-induced error.

Complex cervical anatomy frequently impedes precise matrix adaptation, elevating the risk of interfacial gaps and subsequent microleakage—particularly within the subgingival regions required for DME.^21,40
^ This study utilized maxillary premolars specifically to address these morphological challenges. The mesial surfaces of these teeth typically exhibit pronounced developmental concavities, whereas the distal surfaces are characterized by a more convex morphology.^23^ Such anatomical variations present significant hurdles to achieving a hermetic cervical seal. To address these issues, a silicone-based “sulcus simulation” was implemented to provide lateral support, thereby facilitating tight matrix adaptation against the tooth substrate. The absence of statistically significant differences between the mesial and distal positions in this study suggests that optimal matrix stabilization enables a precise seal even within deep concavities, effectively preventing gap formation at the restoration–tooth interface. These findings underscore the premise that meticulous matrix application is a critical clinical determinant for ensuring consistent marginal integrity, regardless of the inherent proximal concavity geometry.

A significant clinical challenge in DME is the substantial irradiance attenuation caused by the increased distance between the light-curing unit tip and the gingival floor, which often exceeds 5–8 mm. This distance typically risks an inadequate degree of conversion at the critical gingival margin.^11^ In the present study, a dual-layer strategy utilizing bulk-fill composites was implemented to mitigate this concern. The enhanced translucency of these materials ensures a sufficient depth of cure, even under conditions of attenuated light intensity. Furthermore, the use of a flowable composite for the initial layer enhanced adaptability to the complex subgingival margins.^2^ The favorable rheological properties of the flowable resin facilitate superior wetting and internal adaptation within the deep proximal box, effectively sealing challenging anatomical margins.

Beyond the tooth–restoration interface, the longitudinal success of DME is fundamentally contingent upon the interfacial integrity between superimposed restorative layers. In this study, both micro-CT and dye penetration analysis showed the absence of microleakage at the interface between the initial DME foundation and the subsequent definitive restoration across all groups. These findings provide significant clinical reassurance, suggesting that the re-preparation of the DME layer does not constitute a mechanical or biological limitation. Rather, the re-prepared surface of the primary increment maintains sufficient residual unsaturation or forms a high-energy surface to ensure a seamless bond with the subsequent composite layer.^21,29
^ Therefore, these results validate the reliability of the DME procedure, confirming that the incremental transition does not represent a weak link or a site for potential failure.

The present study employed micro-CT analysis and methylene blue dye penetration as two methodologically distinct and deliberately complementary approaches to interfacial evaluation. This dual-method design was constructed to provide a multi-scale characterization of interfacial behavior, with each method contributing non-overlapping information at its respective scale of resolution and sensitivity.

A fundamental limitation of micro-CT analysis in adhesive dentistry is the inability to reliably discriminate the thin adhesive film from the adjacent dentin substrate based on grayscale attenuation values alone. Contemporary bonding systems lacking radiopaque fillers produce adhesive layers with radiodensity values that overlap substantially with surrounding dentin,^27^ rendering threshold-based segmentation unreliable.^5,45
^ Compounding this contrast limitation, at a voxel size of 10 µm, adhesive films measuring 1–5 µm in thickness^9^ and nascent interfacial gaps are subject to the partial volume effect, whereby boundary voxels spanning two structures of differing density are assigned intermediate grayscale values, producing artificially blurred margins at the adhesive–dentin interface and precluding accurate segmentation.^35^ This technological threshold explains the apparent discrepancy whereby longitudinal micro-CT registration demonstrated continuous interfacial adaptation with no observable differences in gap width, while the subsequent dye penetration assessment detected microleakage at the sub-voxel scale.

Methylene blue dye penetration addresses precisely the scale of interfacial failure that micro-CT cannot resolve. With a molecular diameter of approximately 1.3–1.5 nm, the tracer penetrates interfacial gaps, nanoleakage channels, and degraded resin-infiltrated zones orders of magnitude smaller than the micro-CT voxel resolution threshold, operating as a direct functional indicator of interfacial micropermeability, the clinically relevant pathway through which bacteria, fluid, and dissolved ions access the tooth–restoration interface and initiate secondary caries or adhesive degradation.^1,41
^ Accordingly, the absence of macroscopic gaps on micro-CT and the microleakage detected by dye penetration are expected to co-exist: they reflect distinct, non-overlapping phenomena at different dimensional scales and together provide a complete multi-scale characterization of interfacial performance.

Despite its widespread application in interfacial sealing research, methylene blue dye penetration may potentially overestimate microleakage due to specific physicochemical characteristics of the tracer. The molecular dimensions of methylene blue are several orders of magnitude smaller than the average diameter of oral bacteria (1–4 µm)^1,42
^ or dentinal tubules (0.5–1.0 µm),^24^ meaning the dye can infiltrate spaces that are biologically impermeable to pathogenic microorganisms. Furthermore, the high adsorption affinity of methylene blue for dental substrates, particularly collagen and hydroxyapatite, may lead to artifactual over-penetration.^1,41
^ To minimize the risk, the immersion period in the present study was strictly limited to 12 h, a duration considered sufficient for diagnostic dye penetration without excessive substrate saturation.^1^


Although the present study employed a clinically representative model simulating the gingival sulcus environment, the influence of gingival crevicular fluid effusion during the bonding procedure was not replicated. It would be of considerable scientific merit to develop an experimental model that incorporates fluid seepage dynamics at the bonding interface, more closely approximating the clinical scenario. Additionally, the biological impact of the oral microbiome on the adhesive–dentin interface warrants further investigation, as microbial activity may critically accelerate hybrid layer degradation over time. Equally important, functional masticatory forces constitute a principal determinant of the long-term clinical longevity of adhesive restorations; therefore, assessment of interfacial bond integrity under simulated fatigue loading conditions is strongly warranted. Collectively, future investigations should endeavor to incorporate these multifactorial physiological and mechanical stressors to achieve a more comprehensive and clinically valid evaluation of the long-term prognosis of DME procedures.

## Conclusion

Within the limitations of the present study, the etch-and-rinse mode of universal adhesive (UER) demonstrated the greatest susceptibility to interfacial failure following long-term hydrolytic aging, suggesting that the self-etch mode of universal adhesive represented a more clinically reliable alternative for use in the DME procedure. Critically, proximal box concavity geometry did not significantly influence marginal integrity when optimal matrix stabilization was employed. The combined evidence from macro-scale micro-CT structural analysis and sub-micron scale dye penetration assessment collectively supports the clinical reliability of the DME procedure and the incremental transition interface.

### Acknowledgments

The authors would like to thank the Dental Material Science Research Center and Oral Biology Research Center, Faculty of Dentistry, Chulalongkorn University, for providing the research facilities. We sincerely appreciate Assistant Professor Dr. Soranun Chantarangsu for her valuable guidance in statistical analysis as well as Sasiwimon Thonghong for her micro-CT consultation. This study was supported by the Faculty of Dentistry, Chulalongkorn University and Thammasat University Research Unit in Mineralized Tissue Reconstruction, Thailand. The authors declare that no external funding was obtained for this research.

#### Clinical relevance

The long-term marginal integrity of DME restorations is primarily governed by the both adhesive’s effectiveness and optimal matrix application on challenging root dentin. To ensure a durable seal and resistance to hydrolytic degradation, a self-etch approach is suggested over the etch-and-rinse mode of universal adhesives.

#### Declaration of AI and AI-assisted technologies in the writing process

During the preparation of this work, the authors used Gemini (Google) to improve the language and readability of the manuscript. Following the use of this service, the authors reviewed and edited the content as needed and take full responsibility for the final version of the published article.

#### Conflict of interest

The authors declare that they have no conflicts of interest related to this study. No commercial funding was received, and the authors have no financial or personal relationships with the manufacturers of the materials used in this research that could inappropriately influence or bias the findings.

#### Ethical approval

The research protocol was approved by the Human Research Ethics Committee of the Faculty of Dentistry, Chulalongkorn University (HREC-DCU 2023-069).

## References
